# Monocyte‐derived macrophages: The supplements of hepatic macrophage in *Echinococcus multilocularis* infected mice

**DOI:** 10.1002/iid3.699

**Published:** 2022-09-26

**Authors:** Bin Li, Xinwei Qi, Yumei Liu, Yi Yan, Jiaoyu Shan, Xuanlin Cai, Jie Lv, Xuan Zhou, Tao Yu, Xiumin Ma

**Affiliations:** ^1^ State Key Laboratory of Pathogenesis, Prevention and Treatment of High Incidence Diseases in Central Asia, Clinical Laboratory Center Tumor Hospital Affiliated to Xinjiang Medical University Urumqi China; ^2^ Department of Hepatic Hydatid First Affiliated Hospital of Xinjiang Medical University Urumqi Xinjiang China; ^3^ Clinical Laboratory Center Children's Hospital of Xinjiang Uygur Autonomous Region Urumqi Xinjiang China; ^4^ Basic Medical College Xinjiang Medical University Urumqi Xinjiang China; ^5^ Shandong Institute of Parasitic Diseases Shandong First Medical University & Shandong Academy of Medical Sciences Jining China

**Keywords:** *Echinococcus multilocularis* infection, kupffer cells, monocyte‐derived macrophages

## Abstract

**Background:**

Alveolar echinococcosis is a potentially lethal zoonosis caused by the cestode *Echinococcus multilocularis*. This study is to investigate the dynamic changes of monocytes, macrophages, and related cytokines in animal models of persistent infection of *E. multilocularis*.

**Methods:**

An infection model was established by intraperitoneal injection of a protoscolex suspension. The pathological changes of liver were observed by HE staining. The percentage of Ly6C^hi^ and Ly6C^lo^ Monocytes in peripheral blood was detected by flow cytometry. The distribution and expression of CX3CL1, CX3CR1, iNOS, CD163, and CD11b in the liver were detected by immunohistochemistry. The mRNA expression of tumor necrosis factor‐α (TNF‐α) and Arg1 in the liver was detected by quantitative reverse transcription polymerase chain reaction. The expression of INF‐γ, interleukin‐17 (IL‐17), IL‐4, and IL‐10 in peripheral blood was detected by enzyme‐linked immunosorbent assay.

**Results:**

Hematoxylin‐eosin(HE) staining showed that significant lesions appeared in the later stages of infection in the liver. The proportion of Ly6C^hi^ monocytes in the peripheral blood of the experimental group mice decreased after a brief rise, Ly6C^lo^ monocytes decreased first and then increased. The expression of CX3CL1, CX3CR1, CD11b, CD163, and iNOS in the mice liver of the experimental group was increased. The expression level of TNF‐α and Arg1 mRNA in the liver of the experimental group mice increased. The expression level of IFN‐γ, IL‐17, IL‐4, and IL‐10 increased with the duration of infection.

**Conclusions:**

Monocytes as a supplement to hepatic macrophage, monocytes and kupffer cells may both participate in Th1 and Th2 immune responses by differentiating into M1 or M2 at different stages of *E. multilocularis* infection.

## Graphical abstract

Monocytes and kupffer cells may both participate in Th1 and Th2 immune responses by differentiating into M1 or M2 at different stages of *Echinococcus multilocularis* infection.

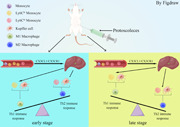

## 1 INTRODUCTION

Echinococcosis refers principally to two serious zoonosis caused by *Echinococcus granulosus* and *Echinococcus multilocularis*, cystic echinococcosis, and alveolar echinococcosis (AE). Both diseases are transmitted through the ingestion of parasite eggs in the feces of the ultimate host, and humans are not necessary intermediate hosts in the life cycles of the two parasites. The life cycle of *E. multilocularis* takes place between canids as the definitive hosts and their prey, small mammals such as rodents, which act as intermediate hosts. Annually there are estimated more than 16,000 new cases worldwide of AE, with 90% of those occurring in China.^1^ In AE patients, 98% of infections occur primarily in the liver, and the mortality rate of untreated or poorly treated patients after diagnosis is about 90% within 10–15 years.^2^ AE lesions can remain asymptomatic for 10–15 years. Clinical symptoms usually appear when a cyst reaches more than 10 cm in diameter in the liver or when more than 70% of the organ volume is occupied by a cyst or cysts.^3^

Macrophages play a leading role in regulating liver homeostasis under physiological and pathological conditions. Resident macrophages of the liver, kupffer cells (KCs), represent a unique cell population. Most of the KCs belong to the self‐sustaining macrophage cell population, whose origin is not in the bone marrow.^4^ KCs play an important role in maintaining homeostasis of the liver through five major functions. These include (i) clearance of cellular debris and metabolic waste, (ii) maintenance of iron homeostasis via phagocytosis of red blood cells (RBCs) and the subsequent recycling of iron, (iii) regulation of cholesterol homeostasis through the production of cholesteryl ester transfer protein, (iv) mediation of antimicrobial defense, and (v) promotion of immunological tolerance.^5^ Blood monocyte‐derived macrophages are a small fraction of macrophages in the liver. According to different estimates, they account for 5%–30% of the total number of liver macrophages.^6^, ^7^ Monocytes are short‐lived mononuclear phagocytes that circulate in the bloodstream and comprise two main subsets that in the mouse are best defined by the Ly6C marker. These cells are known to efficiently extravasate into tissues. Indeed, following recruitment to injured tissue, Ly6C^hi^ monocytes respond to local cues and can critically contribute to the initiation and resolution of inflammatory reactions. The second main murine monocyte subset, Ly6C^lo^ cells, remains in the vasculature, where the cells act as scavengers. Moreover, a major fraction of Ly6C^lo^ monocytes adhere to the capillary endothelium and patrols the vessel wall for surveillance.^8^ Chemokines are cytokines with chemotaxis functions, which are specifically used to regulate the migration of immune cells into damaged or diseased organs. A variety of chemokines and receptors is expressed on the surface of monocytes and macrophages. These chemokines and receptors are involved in migration. The CX3CL1/CX3CR1 pathway affects the recruitment of leukocytes, including monocytes and macrophages, to target areas by affecting the expression of cytokines and chemokines in the active phase of the disease.^9^ KCs and monocyte‐derived macrophages play different or crucial roles in the inflammatory response caused by persistent infection of the liver.^10^ Different types of macrophages synthesize and secrete cytokines such as tumor necrosis factor‐α (TNF‐α), IL‐1b, IL‐6, IL‐17, IL‐4, IL‐12, IL‐18, IL‐10, and IFN‐γ will increase and fully participate in the immune response when they are stimulated.^4^, ^11^, ^12^ Now, two different phenotypes of macrophages were described: one of them called classically activated (or inflammatory) macrophages (M1) and the other alternatively activated (or wound‐healing) macrophages (M2). Both types of macrophages represent opposite ends of a continuum of intermediate phenotypes. M1 macrophages (marked by iNOS, TNF‐α) are generally understood to play a positive role, they are mainly involved in Th1 type immune response, promoting inflammatory response and enhancing the body's ability to kill pathogens. M2 macrophages (marked by CD163, Fizz1, Arg1) are mainly involved in Th2 type immune response and humoral immunity, which can cause relatively weak lethality to the pathogens in the cell, and at the same time strengthen the ability to remove body residual materials and tissue repair.^13^, ^14^

In this study, we used flow cytometry, immunohistochemistry, quantitative reverse transcription polymerase chain reaction (qRT‐PCR), and other methods to detect the dynamic changes of KCs and monocytes in mice during continuous infection of *E. multilocularis*. It was proved that monocyte‐derived macrophages and KCs both secreted Th1 cytokines in the form of M1 to participate in the immune response in the early stage of infection, and secreted Th2 cytokines in the form of M2 to participate in the process of infection in the late stage of infection.

## 2 METHODS

### 2.1 Mice

SPF grade Balb/c mice, female, 80, 6–8 weeks old, provided by the First Affiliated Hospital of Xinjiang Medical University Experimental Animal Science Research Department. All experimental procedures were conducted under strict guidelines and approval of the Institutional Animal Use and Care Committee at Xinjiang Medical University (Approving Number: 20170214‐106).

### 2.2 Grouping and establishment of *E. multilocularis* infection model

Eighty Balb/c mice were randomly divided into two groups, the experimental group (40) and the control group (40). Forty Balb/c mice were inoculated intraperitoneally with *E. multilocularis*, the mice were killed by cervical dislocation, the cysts fluid and the infected tissue were removed aseptically in a 50 ml syringe, respectively. The tissue was ground in a sterile mortar. The ground tissue was sifted with a prepared normal saline solution containing 500 U/ml penicillin and 100 U/ml streptomycin through a 200‐mesh nylon mesh. The filtrate was put into a 50 ml sterile centrifuge tube to make a protoscolex deposit. Finally, the sediments were resuspended in phosphate buffered saline (PBS) to prepare the 4000 protoscoleces/ml suspension. The mice in the experimental group were injected intraperitoneally with 500 μl of protoscolex suspension, and the mice in the control group were injected intraperitoneally with the same volume of saline. They were maintained in an air‐conditioned animal room with a 12‐h light/dark cycle and provided with rodent chow and water.

### 2.3 Specimen collection

Specimens were taken and preserved 2, 8, 30, 90, and 180 days after infection respectively. A total of 1000 μl of blood was collected in a 1.5 ml centrifuge tube from eyes' blood. Approximately, 500 μl was transferred to a blood collection tube with EDTA‐2K and awaited detections by flow cytometry. The rest was left at room temperature for 1 h, centrifuged at 4°C, 3000 r/min for 5 min, and the upper serum obtained after centrifugation was transferred to a new centrifuge tube. The specimens were labeled and frozen in a refrigerator at −20°C until serological detections. After the blood collection, the mice were killed immediately by cervical dislocation and then immersed in 75% alcohol for 5 min. The mice were dissected and observed for intra‐abdominal infection and photographed. The liver of the mice were stripped aseptically and photographed. Part of the livers were fixed in 4% paraformaldehyde fixing solution for 7 days, waiting for subsequent histopathological and immunohistochemical staining detections. The remaining part of the liver was placed in a cryopreservation tube and frozen in liquid nitrogen immediately, then transferred to a −80°C refrigerator for storage, waiting for subsequent qRT‐PCR experiments.

### 2.4 Histopathology

The Paraformaldehyde‐fixed samples were dehydrated in ethanol, cleared with xylene, and embedded in paraffin wax. Sections (4 μm thick) were prepared and stained with hematoxylin and eosin.

### 2.5 Flow cytometry

A total of 500 μl of blood was collected from mice by tube with EDTA‐K2, red cells lysed using RBC lysis buffer (Biolegend), the remaining cells stained with antibodies at 4°C for 30 min, and then the cells were washed with 1% bovine serum albumin wash solution. The cells were suspended in 300 μl of PBS solution and then sorted using a BD LSR II system. PE Rat Anti‐Mouse CD45 (Becton, Dickinson and Company), FITC Rat Anti‐CD11b (Becton, Dickinson and Company), and PerCP‐Cy™5.5 Rat Anti‐Mouse Ly‐6C (Becton, Dickinson and Company) were used. Gated as shown in Supporting Information: Figures 1 and 2. All the data were processed using the FlowJo (Tree Star).

**Figure 1:**
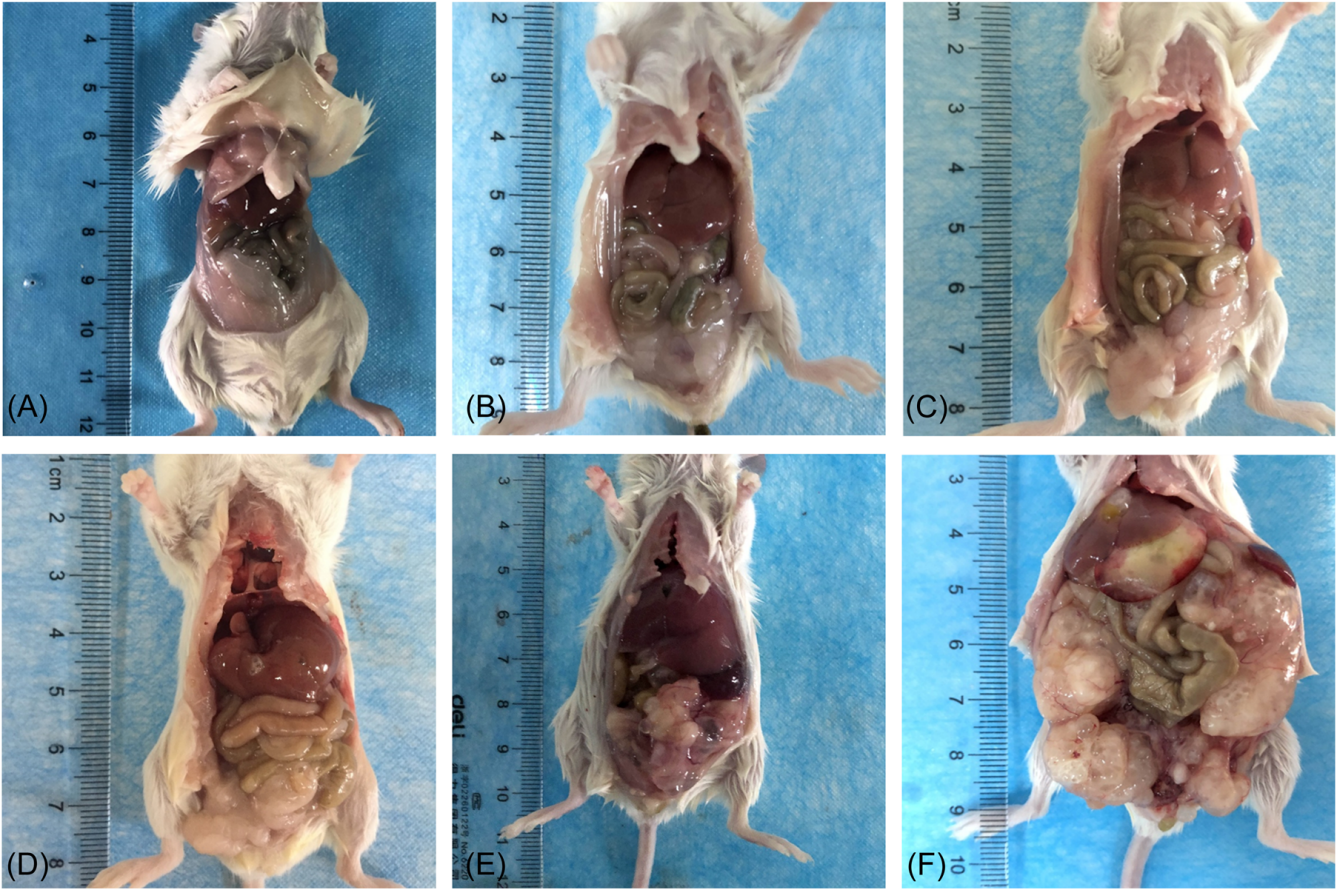
General condition of mice infected with *Echinococcus multilocularis*. (A) Control group; (B) infected after 2 days; (C) infected after 8 days; (D) infected after 30 days; (E) infected after 90 days; (F) infected after 180 days.

**Figure 2:**
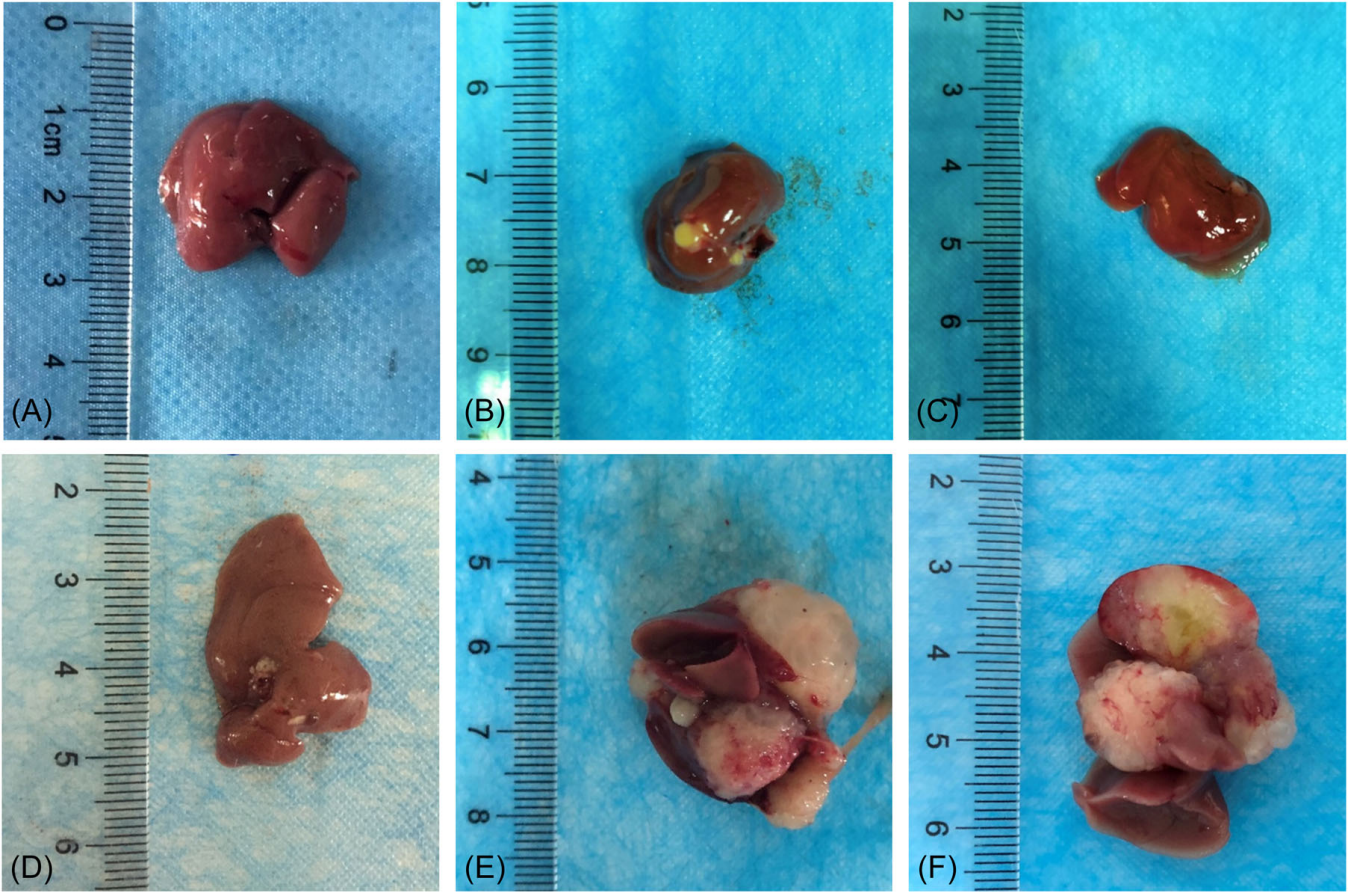
General condition of liver in mice infected with *Echinococcus multilocularis*. (A) Control group; (B) infected after 2 days; (C) infected after 8 days; (D) infected after 30 days; (E) infected after 90 days; (F) infected after 180 days.

### 2.6 Immunohistochemistry

The sections were routinely dewaxed to water, incubated with 3% hydrogen peroxide at room temperature for 10 min, placed in citrate buffer at 95°C for 15 min, and cooled to room temperature. The sections were blocked in goat serum for 30 min and incubated overnight at 4°C with the following primary antibodies: rabbit anti‐CX3CL1 (1:500; Bioss), rabbit anti‐CX3CR1 (1:500; Bioss), rabbit anti‐iNOS (1:400; Bioss), rabbit anti‐CD163 (1:500; Bioss), and rabbit anti‐CD11b (1:4000; Abcam). After being washed in PBS solution, the sections were incubated with the secondary antibodies for 1 h. The following secondary antibodies were used: goat anti‐rabbit IgG horseradish peroxidase (HRP) conjugate (ZSGB‐Bio). After being washed in PBS solution, develop color at room temperature with 3,3'‐diaminobenzidine. The nuclei were stained with hematoxylin. The positive area was counted by an unbiased, blinded investigator using ImageJ software.

### 2.7 RNA isolation and qRT‐PCR

Total RNA of liver samples was isolated using the TRIzol reagent (Invitrogen) according to the manufacturer's instructions and reverse‐transcribed using Prime Script RT Master Mix (RR036A; Takara). Real‐time PCR was performed using SYBR Premix Ex Taq II (RR820A; Takara) in Thermal Cycler Dice™ Real Time System II (BIO‐RAD, IQ5). All PCRs were performed at least twice. The comparative method of relative quantification (2^−ΔΔCt^) was used to calculate the expression level of the target gene normalized to the housekeeping gene glyceraldehyde‐3‐phosphate dehydrogenase. The primers used for qRT‐PCR were listed in Table 1.

**Table 1:** qRT‐PCR using gene primers

Gene	Primers (5′ → 3′)
TNF‐α	Forward: CCCCTTTATCGTCTACTCCTC
	Reverse: GCTGGTAGTTTAGCTCCGTTT
Arg1	Forward: CTCCAAGCCAAAGTCCTTAGAG
	Reverse: AGGAGCTGTCATTAGGGACATC
GAPDH	Forward: AACTTTGGCATTGTGGAAGG
	Reverse: CACATTGGGGGTAGGAACAC

Abbreviations: GAPDH, glyceraldehyde‐3‐phosphate dehydrogenase; qRT‐PCR, quantitative reverse transcription polymerase chain reaction; TNF‐α, tumor necrosis factor‐α.

### 2.8 Enzyme‐linked immunosorbent assay for quantitative detection of INF‐γ and other cytokines

A total of 50 μl samples (serum) and diluted standards were added into wells, and then, 50 μl of biotin conjugate was added into each well. The plates were covered with adhesive film and incubated at room temperature for 2 h on a microplate shaker. Then, the wells were washed six times with a wash buffer. Next, 100 μl of diluted streptavidin‐HRP was added to wells and then incubated at room temperature for 1 h. After six washes, 100 μl of TMB substrate solution was added into each well, incubated at room temperature for 30 min, and then a stop solution was added. The optical density in each well was measured by a microplate reader (Multiskan Spectrum; Thermo Scientific). Cytokine concentrations were calculated by referring to standard curves.

### 2.9 Statistical analysis

Results were analyzed using SPSS software (version 17). Comparisons between groups were made using *χ* ^2^ test/one‐way analysis of variance. Differences were considered significant when *p* < .05.

## 3 RESULTS

### 3.1 General condition of mice

With the prolongation of the feeding time, the weight of the two groups of mice showed an increasing trend, but the increase in the experimental group was more significant (Table 2). The mice of the control group showed no abnormal changes in the liver and abdominal cavity after dissection. The liver texture was soft, smooth, ruddy in color without obvious adhesion. The anatomical position was normal. After dissecting the mice in the experimental group, it was found that as the infection continued, the liver of the mice gradually hardened, the color gradually faded, and the anatomical position gradually deviated from the normal anatomical position. At 30 days after infection, the cysts were seen on the liver, then the cysts gradually increased. The boundary between cysts and tissue was blurred. The cysts were also formed in the abdominal cavity. At the late infection, the abdominal cavity was almost full of cysts (Figures 1 and 2).

**Table 2:** Changes in the weight of mice infected with *Echinococcus multilocularis* in different periods ($\bar{x}$ ± *s*)

Days after infection (d)	*n*	Weight (g)	
		Control group	Experimental group
2	8	21.52 ± 0.65	21.09 ± 1.01
8	8	21.82 ± 0.77	21.93 ± 0.85
30	8	23.65 ± 0.58	24.71 ± 1.03
90	8	24.88 ± 0.44	30.23 ± 2.39*
180	8	28.46 ± 1.02	37.56 ± 3.36**

*Note*: The experimental group was compared with the control group at the same period.

* *p* < .05;

** *p* < .01.

### 3.2 Histological analysis of the liver tissue

**Figure 3:**
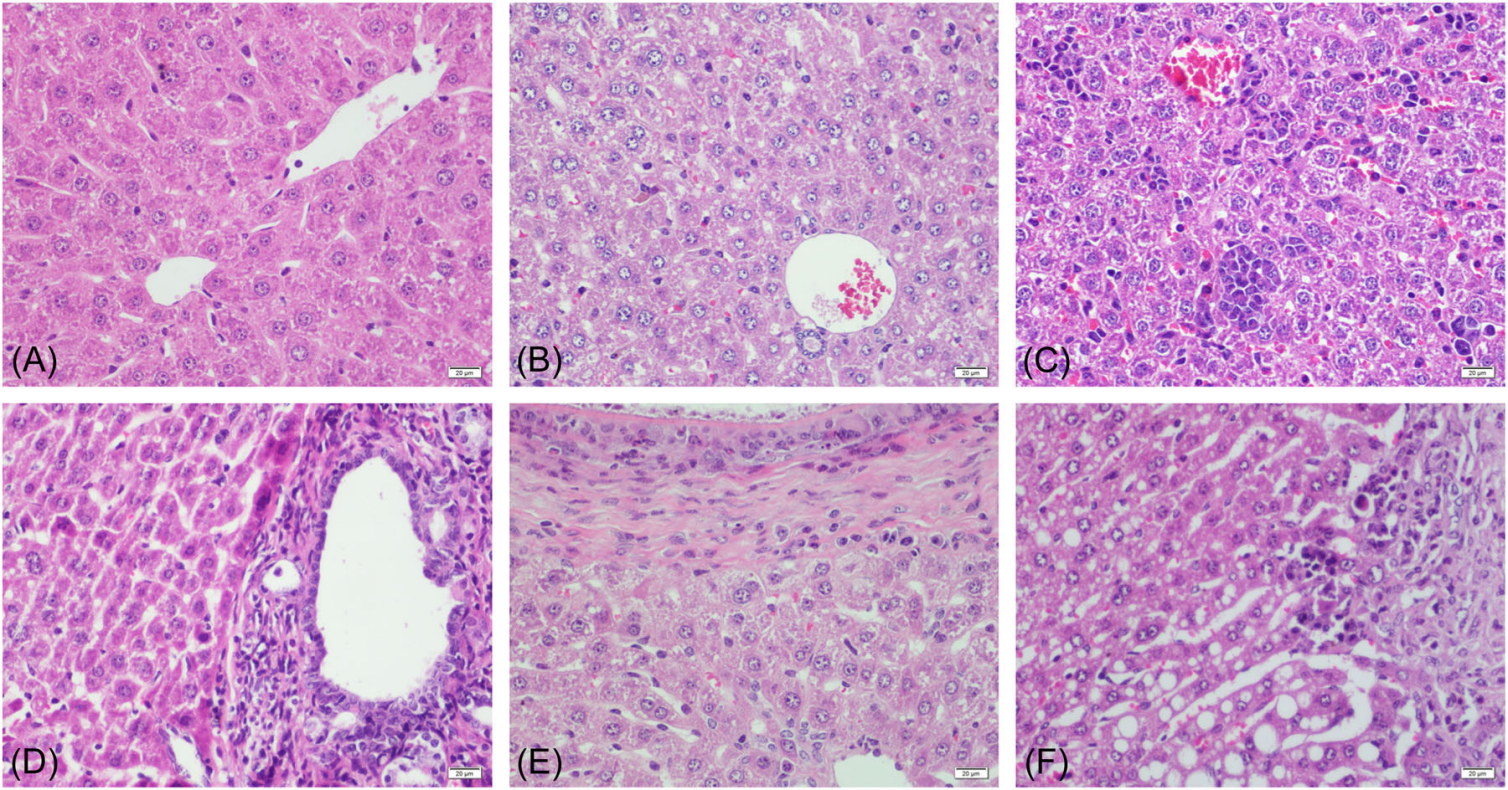
Hematoxylin‐eosin (HE) staining of liver in mice infected with *Echinococcus multilocularis* (×400). (A) Control group; (B) infected after 2 days; (C) infected after 8 days; (D) infected after 30 days; (E) infected after 90 days; (F) infected after 180 days. Scale bar: 20 μm.

**Figure 4:**
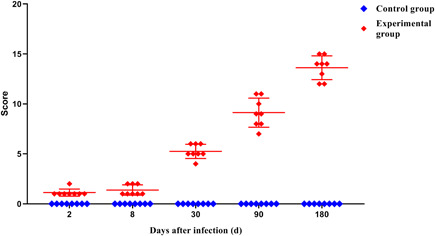
Total score of liver tissue in mice infected with *Echinococcus multilocularis* in different periods

The control group's mice in all periods had normal liver tissue structure and complete hepatic lobules. Hepatocytes were arranged neatly with clear and complete borders. There was no edema, necrosis, and ballooning degeneration in the liver cell. After observing the mice in the experimental group, we found that as the infection continued, the hepatocytes gradually developed from initial edema to ballooning degeneration and steatosis. At 8 days of infection, the mild inflammation in the portal area began to appear, and it became worse with the prolongation of the infection time. At the same time, a small number of focal necrosis could be seen in the hepatic lobules. With the prolongation of infection time, it gradually increased, developed into fusion necrosis or bridging necrosis, and involved many hepatic lobules. In the late stage of infection, a large number of fibroblasts around the lesion proliferated to form a fibrous layer, and a large number of inflammatory cells infiltrated. Normal hepatic lobule structures were no longer visible (Figure 3). According to the Hepatic Infection of *E. multilocularis* Severity Score (Mouse),^15^ we summed up the scores and found that, the longer the time, the more severer the infection (Figure 4).

### 3.3 Detection results of monocyte subsets in peripheral blood

**Figure 5:**
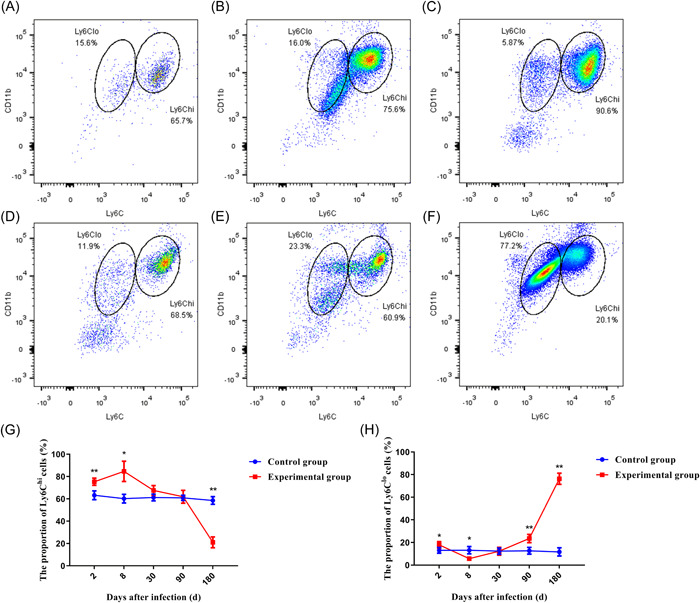
Dynamic changes of the proportion of Ly6C^hi^ and Ly6C^lo^ type monocytes in peripheral blood of mice infected with *Echinococcus multilocularis*. *p < .05, **p < .01. (A) Control group; (B) infected after 2 days; (C) infected after 8 days; (D) infected after 30 days; (E) infected after 90 days; (F) infected after 180 days. (G) Dynamic change of Ly6C^hi^ type monocyte Proportion. (H) Dynamic change of Ly6C^lo^ type monocyte Proportion.

Then we used flow cytometry to detect the proportion of monocyte subsets in the peripheral blood of mice in control and experimental groups in different periods. We found a little change in the proportion of two kinds of monocytes in mice of the control group. In the experimental group, the proportion of Ly6C^hi^ type monocytes gradually increased in the early stage of infection, reached a peak at 8d after infection. Then it began to show a downward trend. At the later stage of infection, it had been significantly lower than the control group at the same time (Figure 5G). For Ly6C^lo^ type monocytes, its proportion rapidly decreases to a minimum after a brief rise in the early stages of infection. Then its proportion began to rise, and at the later stage of the infection, its proportion was already significantly larger than that of the control group during the same period (Figure 5H). The above results showed that in the continuous infection of *E. multilocularis*, the mainly monocyte subsets in peripheral blood transited from Ly6C^hi^ to Ly6C^lo^. Ly6G was not used for labeling in this study, so there may be some neutrophil contamination in the results.

### 3.4 Immunohistochemical results of liver tissue of *E. multilocularis* infection mice

**Figure 6:**
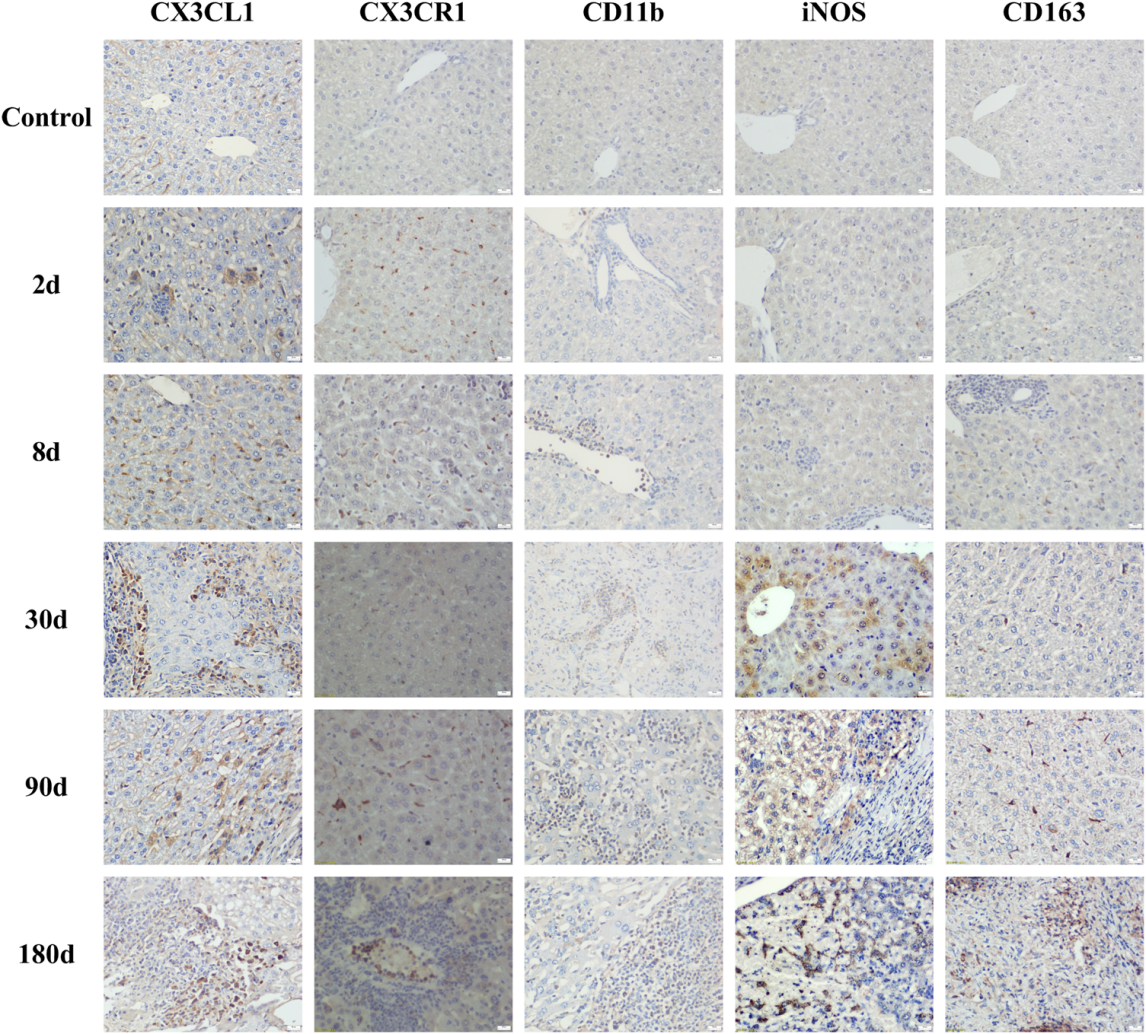
Immunohistochemical results in liver of mice infected with *Echinococcus multilocularis* at different periods (×400). Scale bar: 20 μm.

**Figure 7:**
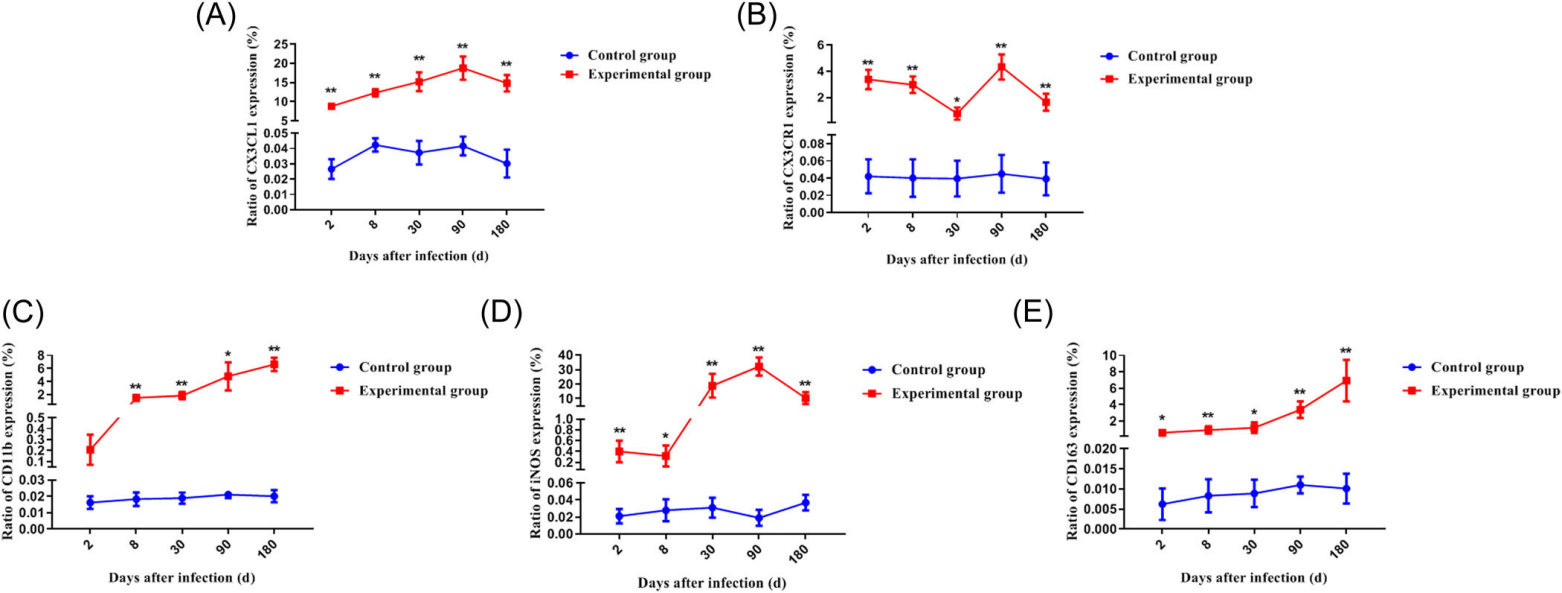
Immunohistochemical positive cells area of liver tissue in mice infected with *Echinococcus multilocularis*. *p < .05, **p < .01. (A) CX3CL1; (B) CX3CR1; (C) CD11b; (D) iNOS; (E) CD163.

Earlier, we observed changes in the proportion of peripheral blood mononuclear cell subsets in infected mice. So did these cells reach the lesion? Subsequently, we used immunohistochemistry to detect neutrophil and monocyte marker, CD11b, related chemokines, and their receptors CX3CL1 and CX3CR1, different types of macrophages markers, iNOS and CD163 (Figure 6). The results showed that all indicators were low and stable in the control group. The expression of chemokine CX3CL1 increased with the prolongation of the infection time. Although the expression level decreased in the late stage of infection, it remained at a high level (Figure 7A). Most of its expression locations were concentrated in inflammatory cell clusters or around lesions. CX3CR1 had a higher expression level than the control group at the early stage of infection. Although the expression level decreased briefly at 30 days after infection, it was still significantly higher than that of the control group in the later period (Figure 7B). The location of its expression gradually concentrated around the lesion with the prolongation of the infection time. For CD11b, its expression increased with the prolongation of infection time (Figure 7C), and almost all of the expression sites were at the inflammatory cell aggregation site. The expression of iNOS that one of the markers of M1 macrophages, increased with the prolongation of the infection time, but the expression level in the late stage of infection was no longer than the intermediate stage (Figure 7D). CD163 is one of the markers of M2 macrophages. The expression of CD163 gradually increased with the prolongation of infection time. Unlike iNOS, the expression of CD163 in the late stage of infection was the highest during the entire infection process (Figure 7E). In summary, our results illustrate the following conclusion, under the role of chemokines and their receptors, both neutrophils and monocytes expressing CD11b were recruited into the liver and concentrated around the lesion at the late stage of infection. Macrophages around the lesion were predominantly M1 and M2 in the early and late stages of infection, respectively.

### 3.5 Detection of different subsets macrophage related genes

**Figure 8:**
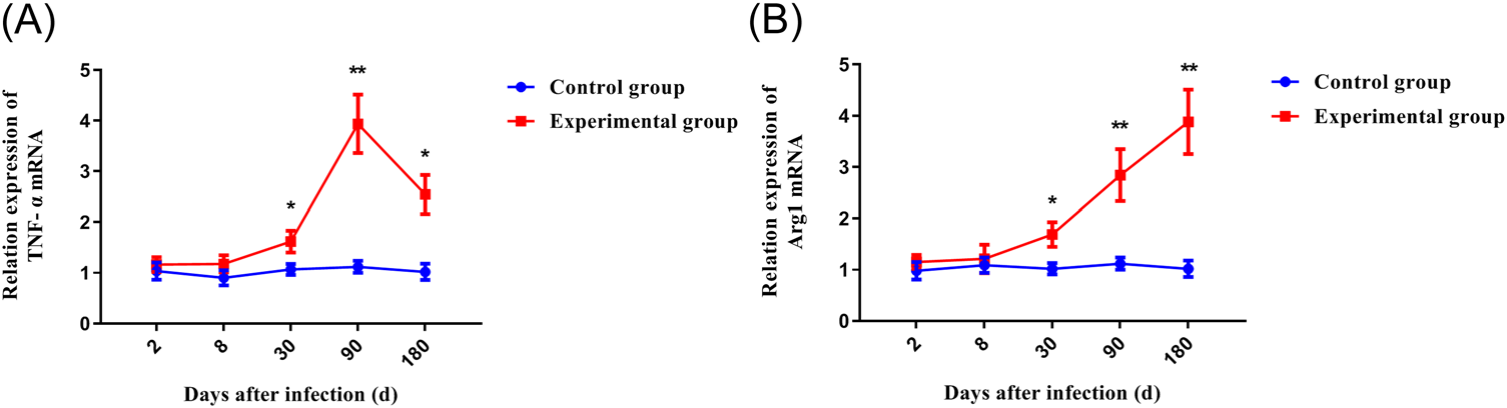
mRNA expression of TNF‐α and Arg1 in liver of mice infected with *Echinococcus multilocularis* at different periods by qRT‐PCR. *p < .05, **p < .01. (A) TNF‐α; (B) Arg1.

After that, to verify the previous conclusions, we carried out the detections of the liver macrophage‐related gene. The results of qRT‐PCR showed that TNF‐α, a marker gene of M1 macrophages, began to increase significantly at 30 days after infection. Its expression reached its peak at 90 days. Although it decreased in the late period, it was still significantly higher than the control group in the same period (Figure 8A). Arg1, one of the marker genes of M2 macrophages, also began to increase significantly at 30 days after infection. But the difference was that it reached a peak in the late stage of infection with the prolongation of the infection time (Figure 8B). The experimental results supported our previous conclusions that the function of macrophages in the liver may undergo a continuous transition from M1 to M2 in the continuous infection of *E. multilocularis*.

### 3.6 Results of detection of macrophages‐related cytokines

**Figure 9:**
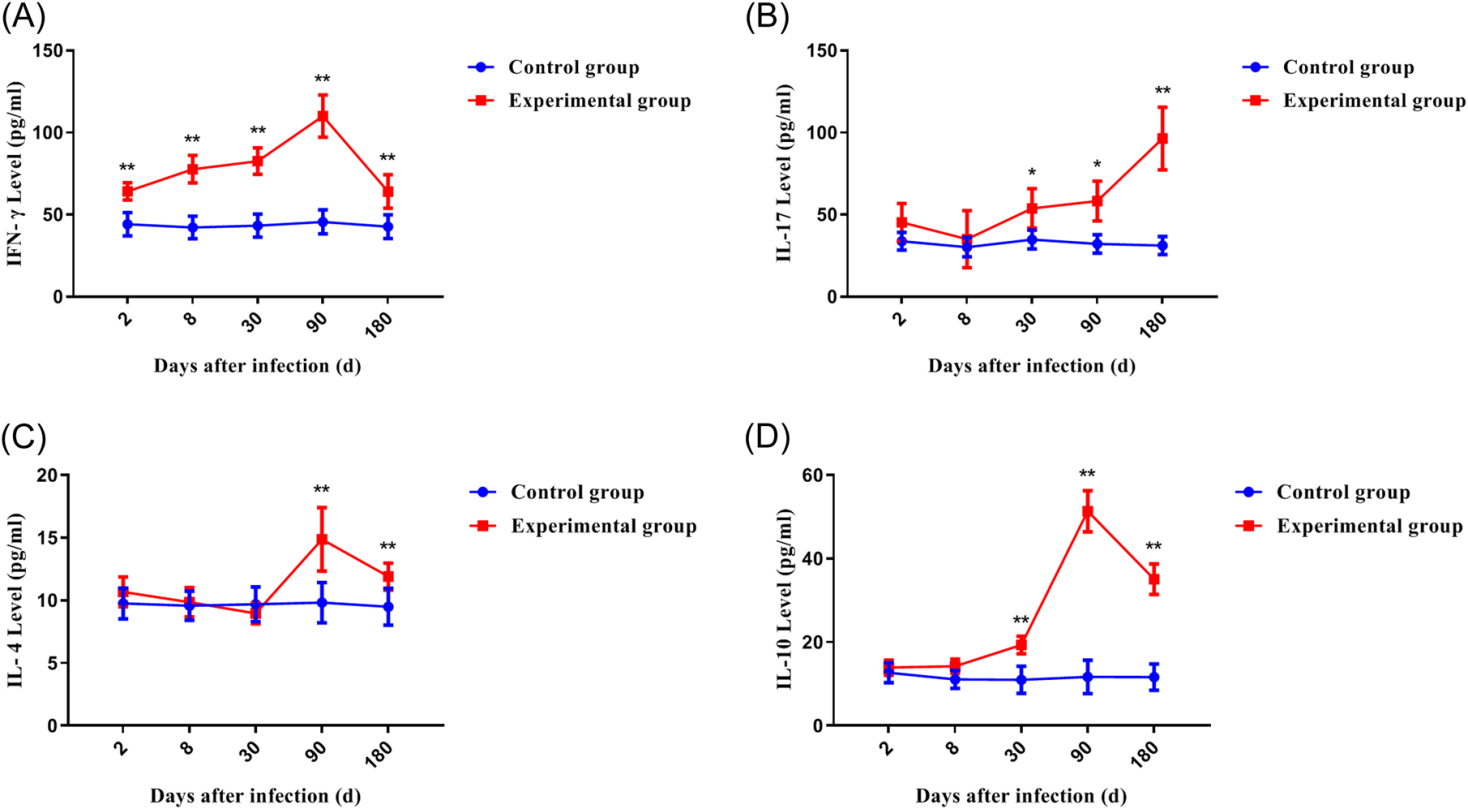
The dynamic changes of mice serum concentration of cytokines infected with *Echinococcus multilocularis*. *p < .05, **p < .01. (A) IFN‐γ; (B) IL‐17; (C) IL‐4; (D) IL‐10.

To explore the immune function of macrophages that have been polarized at different stages of infection, we detected several cytokines associated with different types of macrophages. First, we detected changes in the expression of IFN‐γ in peripheral blood. Its expression trend was similar to that of M1‐type macrophages (Figure 9A). Both of them increase with the duration of infection and begin to decline in the later stages of infection. Then, we detected changes in the expression of IL‐17. During the entire infection process, its expression continued to increase with the prolongation of the infection time, reached a peak in the late stage (Figure 9B). Finally, we continuously detected two cytokines, IL‐4 and IL‐10, and their expression trends were similar. When the infection progressed to 90 days, their expression reached a peak and gradually decreased in the late stage of infection. The difference was that IL‐10 was significantly higher than the control group at 30 days after infection, but IL‐4 was not (Figure 9C,D). This was the difference between the two cytokines. Our results indicate that in the early stage of persistent infection with *E. multilocularis* infection, Th1 immune response represented by IFN‐γ may play a major and important role. In the late stage of infection, maybe Th2 cytokines with higher expression were playing a major role. Therefore, no matter in the early or late stage of infection, macrophages of different subsets may activate and participate in the mice's immune regulation by relevant cytokines, and played an indispensable role.

## 4 DISCUSSION

Echinococcosis is a serious and near‐cosmopolitan disease that continues to be a significant public health issue, with western China being the area of highest endemicity for AE.^3^ The difference in the host's different immune states is an important factor influencing different outcomes of this disease. Therefore, in actual clinical work, the main direction is still to adjust the host's immune status in time, thereby affecting or even changing the occurrence and development of the disease.

As an important part of the body's immune defense mechanism, the function and phenotype of macrophages are highly heterogeneous, depending on its source and type of polarization. Characteristic M1 pro‐inflammatory profile is beneficial for pathogens/tumor elimination but is detrimental for the wound healing process. On the other hand, M2 anti‐inflammatory profile improves chronic inflammatory diseases and regeneration. It has been reported that bone marrow‐derived macrophages adapt to the needs and burdens of the liver under inflammatory conditions, while KCs, liver macrophages that reside in tissues, adapt to the normal function of the liver.^16^ When the liver is damaged, KCs and other liver intrinsic cells will recruit a large number of mature monocytes in the blood circulation to migrate to the damaged site through the action of chemokines and their receptors, and differentiate into different types of macrophages according to different liver microenvironments. For example, the expression of Ly6C^lo^ in recovery macrophages observed during the resolution of mice liver fibrosis was derived from Ly6C^hi^ peripheral blood monocytes and had the characteristics of M1 and M2 macrophages.^17^ Monocyte‐derived macrophages will differentiate into the types that play a positive role in the inflammatory response and aggravates liver damage. At the same time, they will also develop toward a phenotype that promotes tissue repair after liver damage. After selective depletion of KCs with clodronate liposomes or inhibition of monocyte infiltration with CCR2^−/−^ mice, KCs, and inflammatory Ly6C^hi^ monocytes were found to aggravate amebic liver abscess.^18^ In inflammatory response syndrome of liver injury caused by African trypanosome infection, Ly6C^lo^ macrophages can inhibit the pro‐inflammatory function of Ly6C^hi^ monocytes, and differentiate Ly6C^hi^ monocytes into anti‐inflammatory macrophages.^19^

In this study, mice were injected intraperitoneally with protoscoleces to make a model. After the mice were killed, the infection situation in the abdominal cavity of the mice was first observed. We found that with the prolongation of the modeling time, the number and volume of cysts scattered in the abdominal cavity of mice and the liver increased, the amount of sac fluid increased, and the adhesion of cysts to surrounding tissues gradually increased. Squeezing of giant cysts disengages the tissue from its normal position and affects its normal function. The results of HE staining showed that with the progress of *E. multilocularis* infection, the initial focal necrosis in the hepatic lobules gradually developed into fusion or even bridging necrosis. The infiltration of inflammatory cells in the mesial area gradually worsened, and in the late stage of infection, no obvious hepatic lobular structure has been seen, and even part of the lobular structure has disappeared. After that, the infection status of the liver tissues of mice infected with *E. multilocularis* was scored, and the results showed that the total score increased with the prolongation of the infection time. However, the scoring criteria used in this study are semi‐quantitative rather than quantitative. The scores represent only the type of certain pathological patterns and are not true measurements. Therefore, different observers use the same scoring criteria can lead to different conclusions because of their experience or differences in prejudice. Therefore, a more detailed description of the histological morphology can help improve the scoring criteria to play a more detailed role.

After determining the infection status of the mice, we immediately detected the change in the proportion of monocytes in different subsets of peripheral blood by flow cytometry. The results showed that the monocytes in the peripheral blood in the early stage of infection were mainly Ly6C^hi^ type, but in the late stage, they were mainly Ly6C^lo^ type. These monocytes which differentiate into different subsets at different times were affected by chemokines and their receptors. And they were recruited around the lesions of liver tissue. KCs and these monocytes were recruited to the liver mainly exerted their immune function in the form of M1 macrophages in the early stages of infection, but in the later stages, most of them had differentiated into M2 macrophages that were involved in subsequent immune responses. But there is an interesting phenomenon in our results, the expression trends of chemokines and their receptors are not exactly the same. The reason may be that the two indicators at different stages of infection are caused by the expression of different positive cells. First, CX3CL1 has two types: soluble and bound. Second, CX3CL1 positive cells include not only monocytes and macrophages, but also myofibroblasts and endothelial cells. However, CX3CR1 positive cells in mouse liver are mainly macrophages.^20^

Although many cytokines are expressed by more than just macrophages (e.g., TNF‐α). However, the combination of immunohistochemical results and qRT‐PCR results indicated that in the early stage of *E. multilocularis* infection, markers of M1 in the liver were significantly elevated. At this time, M1 may exert a pro‐inflammatory function. In the late stage of infection, M2 markers were significantly upregulated in the liver. At this stage, M2 may perform an anti‐inflammatory and tissue repair role. Therefore, in combination with the previous results of this study, we have reason to believe that in the early stage of *E. multilocularis* infection, a large number of Ly6C^hi^ monocytes in peripheral blood were recruited to the liver, and played an immune role in the form of M1 macrophages together with KCs. In the late stage of infection, a predominant number of Ly6C^lo^ monocytes were recruited into the liver tissue, and it acted as M2 macrophages together with KCs.

Finally, we preliminary explored how different subsets of macrophages are involved in the entire process of *E. multilocularis* infection. We selected several cytokines associated with macrophages, IFN‐γ, IL‐17, IL‐4, and IL‐10. The activation of macrophages and the functions they produce after activation depend on the action of these cytokines.^21^ IFN‐γ was one of the most representative cytokines of Th1 immune response. In contrast, as the most effective antagonist of IFN‐γ, IL‐4 was also selected by us. Then we added one of the representative factors of Th2 immune response, IL‐10. IL‐17 was chosen because it was a recently discovered new type of pro‐inflammatory cytokine. The change in IFN‐γ in the serum of the experimental group mice further proved that during the process of *E. multilocularis* infection, the body's immune type trend is dominated by the early Th1 and gradually transitions to the late Th2 which plays the main function. When the early expression of IFN‐γ is increased, KCs can be activated to promote them to inflammatory sites or pathogenic sites, upregulate the secretion of inflammatory molecules, and enhance the inflammatory response.^22^ However, when Th1 type immune cells had an excessively strong inflammatory response, a higher concentration of IFN‐γ could induce CD4^+^ CD25^+^ T cells to differentiate into CD4^+^ CD25^+^ Tregs, to inhibit the inflammatory response of disorders and reduce body damage.^23^ This may be the reason why IFN‐γ is still maintained at a higher concentration in the late stage of infection. Throughout the infection process, IL‐17 had maintained an upward trend. In the early stages of infection, it may be due to its synergy with other pro‐inflammatory cytokines such as IFN‐γ. However, at the late stage of infection, high levels of IL‐17 may be closely related to liver fibrosis and cirrhosis that during chronic liver disease.^24^ IL‐4, as an important anti‐inflammatory factor, can down‐regulate the effects of IFN‐γ on pathogen killing and tissue damage. And it can down‐regulate the secretion of Th1 cytokines, inhibit Th1 type immune function, and strengthen Th2 cytokine expression to promote the formation of immune tolerance. This is why high concentrations of IL‐4 appear and produce effects in the later stages of infection.^25^ As one of the cytokines overexpressed by M2 macrophages, IL‐10 also has a higher concentration in the late stage of infection. Although high concentrations of IL‐10 helped to suppress inflammatory reactions and repair damaged tissues, they also promoted the formation of immune tolerance in the body and helped pathogens escape the body's immune response.^26^, ^27^ Although there are other immune cells that secrete the above cytokines, our results suggest macrophages are likely to be involved in the *E. multilocularis* infection process.

*E. multilocularis* infection was a long process, and as the prolongation of infection it became a chronic disease gradually, the treatment method would become single, the disease would easily recur, and it would be difficult to cure completely. Therefore, finding a way to prevent chronic infection will be a feasible idea for treating AE. The immune tolerance mechanism formed by the body at the late stage of infection is closely related to the long‐term coexistence of *E. multilocularis* and the host. The imbalance of cellular immunity and humoral immunity caused by the imbalance of Th1 and Th2 is one of vital importance role in this progress. Due to the strong plasticity and functional heterogeneity of macrophages, monocyte‐derived macrophages and KCs were kept to participate in the development of Th1 and Th2 immune functions at the same time. However, there were no clear conclusions about specific functions and mechanisms. Just like we do not know what kind of cell mainly produces which kind of cytokine. But we have reasons to believe that in the future, monocyte‐derived macrophages to supplement depleted KCs to help to treat AE will be a feasible idea.

## 5 CONCLUSION

In a summary, our research indicates that monocytes as a supplement to hepatic macrophage, monocytes and KCs may both participate in Th1 and Th2 immune responses of the body by differentiating into M1 or M2 at different stages of *E. multilocularis* infection.

## AUTHOR CONTRIBUTIONS

Xiumin Ma and Tao Yu conceived and designed the study. Bin Li and Xinwei Qi drafted the manuscript. Xiumin Ma and Bin Li critically revised the manuscript. Bin Li, Yumei Liu, Yi Yan, Jiaoyu Shan, and Xuanlin Cai conducted experimental work. Xinwei Qi, Jie Lv, and Xuan Zhou interpreted data. All the authors read and approved the final manuscript.

## CONFLICT OF INTEREST

The authors declare no conflict of interest.

## Supporting information

Supporting information.

## Data Availability

The data sets used and/or analyzed during the current study are available from the corresponding author on reasonable request.
