# Supplementary material for: Monocyte‐derived macrophages: The supplements of hepatic macrophage in Echinococcus multilocularis infected mice
Source: Immun Inflamm Dis. 2022 Sep 26;10(10):e699. doi: 10.1002/iid3.699 (PMC9511960; doi:10.1002/iid3.699)
Supplement: Supplementary file 1 — Supporting information. [file IID3-10-e699-s001.docx]

Supplementary figures:


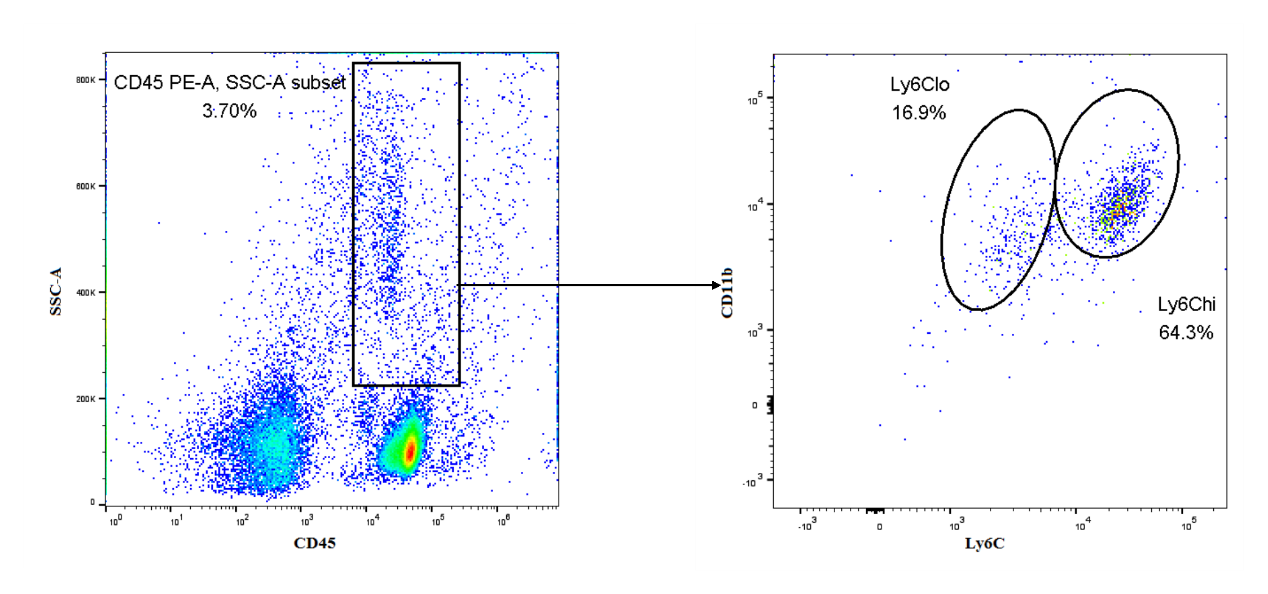
Fig.1 The gating strategy for different subset of monocytes


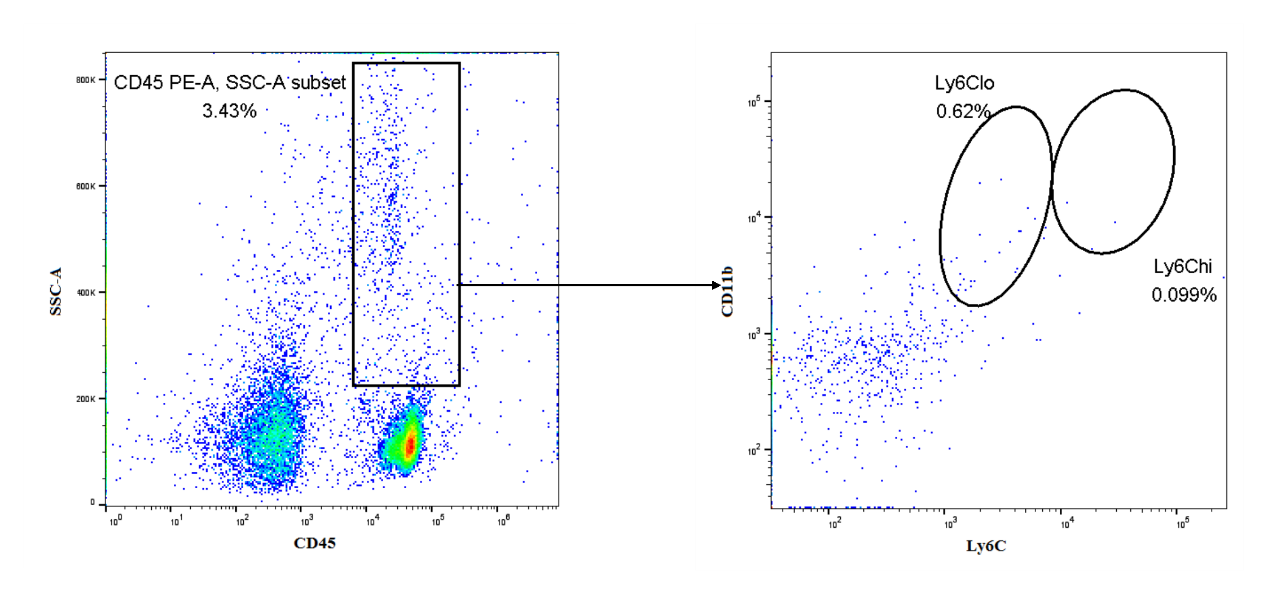
Fig.2 The gating strategy for different subset of monocytes with CD45 labeled only
